# Supplementary material for: Practices and preferences for HIV testing and treatment services amongst partners of transgender women in Lima, Peru: An exploratory, mixed methods study
Source: PLoS One. 2024 Jul 9;19(7):e0306852. doi: 10.1371/journal.pone.0306852 (PMC11232998; doi:10.1371/journal.pone.0306852)
Supplement: S1 Annex — (DOCX) [file pone.0306852.s001.docx]

**S1: Survey questions administered to study participants**

1. **DEMOGRAPHICS**

In the first part of this survey, we would like to ask you some general questions to help us understand who you are.

1. How many years old are you?
   1. *Enter your age in years:* ______
2. What is the highest level of education that you have completed? *Mark only one option.*
   1. Did not complete primary school
   2. Primary school
   3. Secondary school
   4. Post-secondary vocational school
   5. Undergraduate university
   6. Graduate school (e.g. master’s, doctoral)
3. Who do you live with? *Mark all that apply.*
   1. Parent(s)
   2. Sibling(s)
   3. Grandparent(s)
   4. Aunt(s)/Uncle(s)
   5. Cousin(s)
   6. Friend or other roommate
   7. Long-term partner
   8. I live by myself
   9. Other: ____________
4. What district do you currently live in?
   1. Ate
   2. Barranco
   3. Breña
   4. Callao
   5. Chorrillos
   6. Comas
   7. El Agustino
   8. Independencia
   9. Jesús María
   10. La Molina
   11. La Victoria
   12. Lima (Cercado)
   13. Lince
   14. Los Olivos
   15. Magdalena del Mar
   16. Miraflores
   17. Pueblo Libre
   18. Puente Piedra
   19. Rímac
   20. San Borja
   21. San Isidro
   22. San Juan de Lurigancho
   23. San Juan de Miraflores
   24. San Luis
   25. San Martín de Porres
   26. San Miguel
   27. Santa Anita
   28. Santiago de Surco
   29. Surquillo
   30. Villa El Salvador
   31. Villa María del Triunfo
   32. Other ______ (*write in the name of the district*)
5. What is your nationality?
   1. Peruvian
   2. Venezuelan
   3. Other: ____________
6. What is your current employment status?
   1. Working full-time
   2. Working part-time
   3. Unemployed
7. What was your income last month, in soles?
   1. S/. 0
   2. S/. 1 – S/. 749
   3. S/. 750 – S/. 1000
   4. S/. 1001 – S/. 1500
   5. More than S/. 1500
   6. I prefer not to respond to this question
8. What was your sex assigned at birth?
   1. Male
   2. Female
9. What is your current gender identity?
   1. Man
   2. Woman
   3. Non-binary person
   4. Other: ____________
10. **SEXUAL BEHAVIORS**

In this next section, we will ask questions that have to do with your sexual behaviors. Some questions will ask about sexual behaviors within a specific time period, which will be bolded and underlined for clarity, and other will not specify any time period. Those questions that do not specify a time period will be asking about your sexual behaviors in general. All information is confidential and you have the option to stop taking the survey at any point with no penalty if you feel uncomfortable.

1. What is/was your relationship with the transgender woman who referred you? *Only to be asked for persons who indicated sex assigned at birth was “male” and current gender identity is “man.”
   1. She is a friend/not a sexual partner
   2. She is a stable partner/spouse
   3. She is a casual partner/friends-with-benefits
   4. She is a punto/one-time partner (we had sex once and did not exchange money, goods or services in exchange for the sex)
   5. She is a client partner (I sold sex to her)
   6. She is a partner from whom I purchased sex (she sold sex to me)
2. Who are you attracted to sexually?
   1. Cisgender men – a person who was assigned male sex at birth and who identifies as a man
   2. Cisgender women – a person who was assigned female sex at birth and who identifies as a woman
   3. Transgender men – a person who was assigned female sex at birth and who identifies as a man
   4. Transgender women – a person who was assigned male sex at birth and who identifies as a woman
3. With whom do you have sex?
   1. Cisgender men – a person who was assigned male sex at birth and who identifies as a man
      1. Yes
         1. How many of this type of partner have you had in the last 3 months?
            1. Free text: _______
      2. No
   2. Cisgender women – a person who was assigned female sex at birth and who identifies as a woman
      1. Yes
         1. How many of this type of partner have you had in the last 3 months?
            1. Free text: _______
      2. No
   3. Transgender men – a person who was assigned female sex at birth and who identifies as a man
      1. Yes
         1. How many of this type of partner have you had in the last 3 months?
            1. Free text: _______
      2. No
   4. Transgender women – a person who was assigned male sex at birth and who identifies as a woman
      1. Yes
         1. How many STABLE partners have you had in the **last 3 months** that were transgender women? __________
            1. *If answer is not 0: Have you ever exchanged money, goods, services or a place to stay in exchange for sex with any of these partners?

Yes

No

- - - 1. How many casual/friends-with-benefits partners have you had in the **last 3 months** that were transgender women?
         1. *If answer is not 0: Have you ever given any of these partners money, goods, services or a place to stay in exchange for sex?

Yes

No

- - - 1. How many punto/one-time partners have you had in the **last 3 months** that were transgender women? *This is someone that you only had sex with one time, but did not pay for sex.* __________
      2. How many client partners have you had in the **last 3 months** that were transgender women? *This is someone that you sold sex to in exchange for money, goods, services or a place to stay.* __________
      3. How many transgender women partners have you purchased sex from in the **last 3 months**? *This is someone that you bought sex from in exchange for money, goods, services or a place to stay.* __________
    1. No

Now we will ask you a few questions regarding your condom use with each partner type in the **last 30 days**. When answering these questions, please consider **ALL GENDERS** of each partner type that you have had in the **last 30 days.** If you did not have this type of partner in the last 30 days, please indicate that by selecting “No.”

1. Have you had any **stable partners** in the **last 30 days**?
   1. Yes

The next series of sub-questions will have to do with specific sex acts that you completed with **stable partners** in the **last 30 days**.

- - 1. Did you have **receptive anal sex** with any stable partners in the **last 30 days**? *Receptive anal sex is defined as when your partner puts their penis in your anus.*
       1. Yes
          1. How many times did you not use a condom while having **receptive anal sex** with any stable partners in the **last 30 days**? ____________
       2. No
    2. Did you have **insertive anal sex** with any stable partners in **the last 30 days**? *Insertive anal sex is defined as when you put your penis in your partner’s anus.*
       1. Yes
          1. How many times did you not use a condom while having **insertive anal sex** with any stable partners in the **last 30 days**? ____________
       2. No
    3. Did you have **vaginal sex** with any stable partners in the **last 30 days**?
       1. Yes
          1. How many times did you not use a condom while having **vaginal sex** with any stable partners in the **last 30 days**? ____________
       2. No
  1. No

1. Have you had any **casual partner/friends-with-benefits** in the **last 30 days**?
   1. Yes

The next series of sub-questions will have to do with specific sex acts that you completed with **casual partner/friends-with-benefits** in the **last 30 days**.

- - 1. Did you have **receptive anal sex** with any casual partner/friends-with-benefits in the **last 30 days**? *Receptive anal sex is defined as when your partner puts their penis in your anus.*
       1. Yes
          1. How many times did you not use a condom while having **receptive anal sex** with any casual partner/friends-with-benefits in the **last 30 days**? ____________
       2. No
    2. Did you have **insertive anal sex** with any casual partner/friends-with-benefits in **the last 30 days**? *Insertive anal sex is defined as when you put your penis in your partner’s anus.*
       1. Yes
          1. How many times did you not use a condom while having **insertive anal sex** with any casual partner/friends-with-benefits in the **last 30 days**? ____________
       2. No
    3. Did you have **vaginal sex** with any casual partner/friends-with-benefits in the **last 30 days**?
       1. Yes
          1. How many times did you not use a condom while having **vaginal sex** with any casual partner/friends-with-benefits in the **last 30 days**? ____________
       2. No
  1. No

1. Have you had any **puntos/one-time partners** in the **last 30 days**? *Punto/one-time partners are partners that you had sex with one time and did not exchange money, goods or services in exchange for sex.*
   1. Yes

The next series of sub-questions will have to do with specific sex acts that you completed with **puntos/one-time partners** in the **last 30 days**.

- - 1. Did you have **receptive anal sex** with any punto/one-time partners in the **last 30 days**? *Receptive anal sex is defined as when your partner puts their penis in your anus.*
       1. Yes
          1. How many times did you not use a condom while having **receptive anal sex** with any puntos/one-time partners in the **last 30 days**? ____________
       2. No
    2. Did you have **insertive anal sex** with any punto/one-time partners in **the last 30 days**? *Insertive anal sex is defined as when you put your penis in your partner’s anus.*
       1. Yes
          1. How many times did you not use a condom while having **insertive anal sex** with any puntos/one-time partners in the **last 30 days**? ____________
       2. No
    3. Did you have **vaginal sex** with any punto/one-time partners in the **last 30 days**?
       1. Yes
          1. How many times did you not use a condom while having **vaginal sex** with any puntos/one-time partners in the **last 30 days**? ____________
       2. No
  1. No

1. Have you had any **client partners** in the **last 30 days**? *Client partners are partners who bought sex from you.*
   1. Yes

The next series of sub-questions will have to do with specific sex acts that you completed with **client partners** in the **last 30 days**.

- - 1. Did you have **receptive anal sex** with any client partners in the **last 30 days**? *Receptive anal sex is defined as when your partner puts their penis in your anus.*
       1. Yes
          1. How many times did you not use a condom while having **receptive anal sex** with any client partners in the **last 30 days**? ____________
       2. No
    2. Did you have **insertive anal sex** with any client partners in **the last 30 days**? *Insertive anal sex is defined as when you put your penis in your partner’s anus.*
       1. Yes
          1. How many times did you not use a condom while having **insertive anal sex** with any client partners in the **last 30 days**? ____________
       2. No
    3. Did you have **vaginal sex** with any client partners in the **last 30 days**?
       1. Yes
          1. How many times did you not use a condom while having **vaginal sex** with any client partners in the **last 30 days**? ____________
       2. No
  1. No

1. Have you had any **partners from whom you purchased sex** in the **last 30 days**?
   1. Yes

The next series of sub-questions will have to do with specific sex acts that you completed with **partners from whom you purchased sex** in the **last 30 days**.

- - 1. Did you have **receptive anal sex** with any partners from whom you purchased sex in the **last 30 days**? *Receptive anal sex is defined as when your partner puts their penis in your anus.*
       1. Yes
          1. How many times did you not use a condom while having **receptive anal sex** with any partners from whom you purchased sex in the **last 30 days**? ____________
       2. No
    2. Did you have **insertive anal sex** with any partners from whom you purchased sex in **the last 30 days**? *Insertive anal sex is defined as when you put your penis in your partner’s anus.*
       1. Yes
          1. How many times did you not use a condom while having **insertive anal sex** with any partners from whom you purchased sex in the **last 30 days**? ____________
       2. No
    3. Did you have **vaginal sex** with any partners from whom you purchased sex in the **last 30 days**?
       1. Yes
          1. How many times did you not use a condom while having **vaginal sex** with any partners from whom you purchased sex in the **last 30 days**? ____________
       2. No
  1. No

The next group of questions has to do with other sexual behaviors you have had in the **last 3 months.**

1. In the **last 3 months**, did you ever have sex with multiple partners at the same time (for example, in an orgy or other type of group sex)?
   1. Yes
      1. How many times in the last 3 months? ____________
   2. No
2. In the **last 3 months**, have you ever used alcohol or drugs before or during sex?
   1. Yes, I have used: (Mark all that apply)
      1. Alcohol
      2. Marijuana
      3. Cocaine
      4. Methamphetamine
      5. Poppers
      6. Ecstasy/MDMA
      7. Heroin
      8. Ketamine
      9. Other drug: ____________
   2. No
3. In the **last 3 months,** how many times have you drank 5 or more drinks on one occasion before or during sex? *Ask only if they marked “alcohol” for previous question*
   1. 0
   2. 1-3
   3. 4-7
   4. 8+
4. **HIV TESTING HISTORY AND SELF-PERCEIVED RISK**

The next section will ask you questions regarding your testing history for HIV, your HIV status, and questions regarding your perceived risk of acquiring HIV. You may decline to provide information regarding your HIV status. Please know that all of your answers will be kept confidential.

1. Have you ever been tested for HIV? *Mark only one option.*
   1. No, I have never been tested for HIV.
   2. Yes, and the last time I had an HIV test it was negative (I don’t have HIV).
      1. When was the last time you had an HIV test? If you can’t remember exactly how long ago this was, give your best estimate.
         1. Within the last 3 months.
         2. Between 3 and 6 months ago.
         3. Between 6 and 9 months ago.
         4. Between 9 and 12 months ago.
         5. More than one year ago.
         6. I prefer not to answer this question.
   3. Yes, and the result was positive (I have HIV).
      1. How long ago were you diagnosed with HIV infection? If you don’t remember exactly how long ago, give your best estimate.
         1. In the last 6 months.
         2. Between 6 and 12 months ago.
         3. Between 1 and 5 years ago.
         4. More than 5 years ago.
         5. I prefer not to answer this question.
      2. Are you currently taking antiretroviral treatment?
         1. No, and I never have taken it.
         2. No, I was taking it before but not now.
         3. Yes, I am currently taking it.
   4. Yes, but I did not get my result (I am not sure if it was positive or negative).
      1. When was the last time you had an HIV test? If you can’t remember exactly how long ago this was, give your best estimate.
         1. Within the last 3 months.
         2. Between 3 and 6 months ago.
         3. Between 6 and 9 months ago.
         4. Between 9 and 12 months ago.
         5. More than one year ago.
         6. I prefer not to answer this question.
   5. I prefer not to answer this question.

*The following questions will be given only to those who did not answer “c” in the previous question*

1. How likely do you think that it is that you will contract HIV over the next year?
   1. I think I already have HIV
   2. Extremely likely
   3. Somewhat likely
   4. Neither likely nor unlikely
   5. Somewhat unlikely
   6. Extremely unlikely
2. How worried are you about contracting HIV?
   1. Extremely worried
   2. Somewhat worried
   3. Neither worried nor not worried
   4. Somewhat not worried
   5. Not at all worried
3. How bothered would you be if you contracted HIV?
   1. Extremely bothered
   2. Somewhat bothered
   3. Neither bothered nor unbothered
   4. Somewhat unbothered
   5. Not at all bothered

For the next two questions, indicate how much you agree with the following statements:

1. “I think it is important to get tested for HIV.”
   1. Strongly agree
   2. Agree
   3. Neither agree nor disagree
   4. Disagree
   5. Strongly disagree
2. “I think I should get tested for HIV.”
   1. Strongly agree
   2. Agree
   3. Neither agree nor disagree
   4. Disagree
   5. Strongly disagree

The remaining three questions in this section will ask you about ways to prevent HIV infection in yourself and/or others.

1. Have you ever heard of PRE-Exposure Prophylaxis (PrEP) for HIV?
   1. Yes
      1. Have you ever taken PrEP? *PrEP consists of taking a pill every day for an indefinite period of time to prevent HIV infection.*
         1. Yes, I am currently taking PrEP.
         2. Yes, I have taken PrEP in the past but am no longer taking it.
         3. No, I have never taken PrEP.
   2. No
2. Have you ever heard of POST-Exposure Prophylaxis (PEP) for HIV?
   1. Yes
      1. Have you ever taken PEP? *PEP consists of taking a combination of pills AFTER a potential HIV exposure for a defined period of 4 weeks.*
         1. Yes, I have taken PEP at least once.
            1. How many times have you taken PEP in your life? _____
         2. No, I have never taken PEP.
   2. No
3. Do you think the following sentence is TRUE or FALSE?

“If a person who has HIV is taking anti-retroviral therapy so that the virus can no longer be detected in their blood, this person cannot transmit the HIV virus to their sexual partners.”

*If you are not sure of the correct answer, respond “I don’t know.”*

- 1. True
  2. False
  3. I don’t know

1. **RELATIONSHIP WITH THE HEALTHCARE SYSTEM**

The final section of this survey will ask you about your history of seeking healthcare services and HIV services.

The first set of questions have to do with your history of seeking healthcare for **ANY HEALTH REASON** at any point in your life.

1. Have you ever sought care for any health reason at any of the following places? *Mark all that apply.*
   1. Government hospital/clinic (EsSalud)
   2. Government hospital/clinic (MINSA)
   3. CERITSS clinics
   4. Private hospital/clinic
   5. Non-governmental organization
2. Thinking about the healthcare services you have accessed in your life, which of the following are reasons that you have gone to these facilities? *Mark all that apply.*
   1. General check-ups
   2. Vaccination/Immunizations
   3. Mild illness (flu-like symptoms, cold, diarrhea, headaches)
   4. Emergency services
   5. Health screening services (mammograms, blood pressure checks, bloodwork)
   6. Surgical services
   7. Sexual health services, including sexually transmitting infections, except for testing or treatment of HIV
   8. HIV testing
   9. Hormone therapy
   10. Other: ____________
3. Have you ever felt as though you were treated unfairly or discriminated against at this location? *for each location in 30*
   1. Yes
      1. Which of the following are reasons that you believe you were treated unfairly?
         1. Because I am a transgender woman.
         2. Because I am a man who has sex with men.
         3. Because I am gay.
         4. Because I have sex with transgender women.
         5. Because I sell sex for money, goods and/or services.
         6. Other: ____________
   2. No
4. Do you have a primary care provider, a doctor that you see regularly for health-related services?
   1. Yes
      1. How comfortable are you with discussing your sexuality (for example, your sexual partners, the types of people you have sex with, the types of sexual activities you have, etc.) with this provider?
         1. Extremely comfortable
         2. Somewhat comfortable
         3. Neither comfortable nor uncomfortable
         4. Somewhat uncomfortable
         5. Extremely uncomfortable
      2. Have you ever had conversations regarding your sexuality (for example, your sexual partners, the types of people you have sex with, the types of sexual activities you have, etc.) with them?
         1. Yes
            1. Who initiated the questions/conversation?

I did.

My doctor did.

- - - - 1. Were you honest with them about your sexuaity?

Yes

No

- - - 1. No
  1. No

1. How comfortable are you with discussing your sexuality (for example, your sexual partners, the types of people you have sex with, the types of sexual activities you have, etc.) with healthcare providers?
   1. Extremely comfortable
   2. Somewhat comfortable
   3. Neither comfortable nor uncomfortable
   4. Somewhat uncomfortable
   5. Extremely uncomfortable
2. Have you had a sexually transmitted infection (other than HIV) in the **last 3 months**?
   1. Yes, I had: *Mark all that apply.*
      1. Syphilis
      2. Gonorrhea
      3. Chlamydia
      4. Genital Warts
      5. Genital Herpes
      6. Abnormal penile discharge (without a specific diagnosis)
      7. Abnormal anal discharge (without a specific diagnosis)
      8. Other: ____________

For the next couple of questions, we are going to ask specific information about which locations, if any, you went to for testing and treatment for any suspected sexually transmitted infections in the past.

1. Have you ever gone to any of the following healthcare facilities for **TESTING** for sexually transmitted infections? *Mark all that apply.*
   1. Government hospital/clinic (EsSalud)
   2. Government hospital/clinic (MINSA)
   3. CERITSS clinics
   4. Private hospital/clinic
   5. Non-governmental organization
   6. Unmarked van from NGO
   7. I have never gone for testing for a sexually transmitted infection
   8. Other:_____________
2. Have you ever gone to any of the following healthcare facilities for **TREATMENT** of sexually transmitted infections? *Mark all that apply.*
   1. Government hospital/clinic (EsSalud)
   2. Government hospital/clinic (MINSA)
   3. CERITSS clinics
   4. Private hospital/clinic
   5. Non-governmental organization
   6. Pharmacy
   7. I have never gone for treatment of a sexually transmitted infection
   8. Other:_____________
3. Have you ever sought treatment for a suspected sexually transmitted infection (i.e. by asking a physician for a prescription, going directly to a pharmacy or having medications delivered) without seeking testing or care at a healthcare facility?
   1. Yes
      1. How many times? ________
   2. No

The following questions have to do with your experience with testing for HIV. *Will not be asked to those who indicated in question 1 of section III that they have never been tested for HIV*

1. Which of the following types of facilities have you ever gone to for HIV TESTING?
   1. Government hospital/clinic (EsSalud)
   2. Government hospital/clinic (MINSA)
   3. CERITSS clinic
   4. Private hospital/clinic
   5. Non-governmental organization (Epicentro, Via Libre)
   6. Community-based health campaign
2. How satisfied were you with your experiences with HIV testing services at this location? *for this question as well as the following two questions, will be asked for each location marked in question 39*
   1. Extremely satisfied
   2. Somewhat satisfied
   3. Neither satisfied nor unsatisfied
   4. Somewhat unsatisfied
   5. Extremely unsatisfied
3. Have you ever felt as though you were treated unfairly or discriminated against at this location? *for each location in 39*
   1. Yes
      1. Which of the following are reasons that you believe you were treated unfairly?
         1. Because I am a transgender woman.
         2. Because I am a man who has sex with men.
         3. Because I am gay.
         4. Because I have sex with transgender women.
         5. Because I sell sex for money, goods and services.
         6. Other: ____________
   2. No

Before proceeding to the next section, we would like to ask you a general question about HIV testing services.

1. In general, what is your interest in taking an HIV test?
   1. Extremely interested
   2. Somewhat interested
   3. Neither interested nor uninterested
   4. Somewhat uninterested
   5. Extremely uninterested

**Testing *only for participants who are HIV negative or unknown***

In the final section of this survey, we are going to ask you questions about specific features of how HIV testing could be offered and which features are important to you when taking an HIV test.

Location

Now we are going to ask you some hypothetical questions about HIV testing services and your interest in different potential locations. Based on the following different locations that HIV testing may be available, please indicate your level of interest given each hypothetical scenario.

If you were going to get an HIV test, how important would it be for you to be able to get a test at …

| **Location** | **Importance** | | | | |
| --- | --- | --- | --- | --- | --- |
| …a government clinic (EsSalud/MINSA)? | Extremely important | Somewhat important | Neither important nor unimportant | Somewhat unimportant | Extremely unimportant |
| …a government STI clinic (CERITSS)? | Extremely important | Somewhat important | Neither important nor unimportant | Somewhat unimportant | Extremely unimportant |
| …a private clinic? | Extremely important | Somewhat important | Neither important nor unimportant | Somewhat unimportant | Extremely unimportant |
| …a non-governmental organization (Epicentro, Via Libre)? | Extremely important | Somewhat important | Neither important nor unimportant | Somewhat unimportant | Extremely unimportant |
| …a community health campaign? | Extremely important | Somewhat important | Neither important nor unimportant | Somewhat unimportant | Extremely unimportant |
| …a mobile HIV testing van? | Extremely important | Somewhat important | Neither important nor unimportant | Somewhat unimportant | Extremely unimportant |
| …a social venue (ex. Bar/club) or plaza (ex. San Martín)? | Extremely important | Somewhat important | Neither important nor unimportant | Somewhat unimportant | Extremely unimportant |
| …a “self-test” for HIV that can be administered at home? | Extremely important | Somewhat important | Neither important nor unimportant | Somewhat unimportant | Extremely unimportant |
| …a men’s health clinic? | Extremely important | Somewhat important | Neither important nor unimportant | Somewhat unimportant | Extremely unimportant |

Convenience

Now we are going to ask you some hypothetical questions about HIV testing services and your interest in different factors that have to do with hours of availability and wait times. Based on the following different times that HIV testing may be available, please indicate your level of interest given each hypothetical scenario.

If you were going to get an HIV test, how important would it be for you…

| **Situation** | **Importance** | | | | |
| --- | --- | --- | --- | --- | --- |
| … to be able to get tested on weekday evenings, after traditional working hours? | Extremely important | Somewhat important | Neither important nor unimportant | Somewhat unimportant | Extremely unimportant |
| … to be able to get tested on weekends? | Extremely important | Somewhat important | Neither important nor unimportant | Somewhat unimportant | Extremely unimportant |
| … to be able to get tested on weekday mornings? | Extremely important | Somewhat important | Neither important nor unimportant | Somewhat unimportant | Extremely unimportant |
| … to be able to get tested on weekday afternoons? | Extremely important | Somewhat important | Neither important nor unimportant | Somewhat unimportant | Extremely unimportant |
| … to not have to wait more than 10 minutes at the location to get tested? | Extremely important | Somewhat important | Neither important nor unimportant | Somewhat unimportant | Extremely unimportant |
| … to schedule an appointment for the test? | Extremely important | Somewhat important | Neither important nor unimportant | Somewhat unimportant | Extremely unimportant |
| … to be able to get tested at a location close to your house? | Extremely important | Somewhat important | Neither important nor unimportant | Somewhat unimportant | Extremely unimportant |
| … to be able to get tested at a location in a neighborhood where you won’t run into anyone you know? | Extremely important | Somewhat important | Neither important nor unimportant | Somewhat unimportant | Extremely unimportant |
| … to be able to get tested at a location that is close to work? | Extremely important | Somewhat important | Neither important nor unimportant | Somewhat unimportant | Extremely unimportant |
| … to be able to get tested at a location that is easily accessible to public transportation? | Extremely important | Somewhat important | Neither important nor unimportant | Somewhat unimportant | Extremely unimportant |

Confidentiality/Privacy

Now we are going to ask you some hypothetical questions about HIV testing services and your interest in different factors that have to do with confidentiality and privacy. Based on the following different situations, please indicate your level of interest given each hypothetical scenario.

How important is it to you that…

| **Situation** | **Importance** | | | | |
| --- | --- | --- | --- | --- | --- |
| … no one makes assumptions about your HIV status because you took an HIV test? | Extremely important | Somewhat important | Neither important nor unimportant | Somewhat unimportant | Extremely unimportant |
| … no one makes assumptions about your sexual identity because you took an HIV test? | Extremely important | Somewhat important | Neither important nor unimportant | Somewhat unimportant | Extremely unimportant |
| ... you do not encounter anyone you know while waiting for or taking the HIV test? | Extremely important | Somewhat important | Neither important nor unimportant | Somewhat unimportant | Extremely unimportant |
| … your HIV status and other health information be kept private? | Extremely important | Somewhat important | Neither important nor unimportant | Somewhat unimportant | Extremely unimportant |
| …the location you test at is not known as a place where LGBTQ+ community goes for HIV testing? | Extremely important | Somewhat important | Neither important nor unimportant | Somewhat unimportant | Extremely unimportant |

Cost

1. What would you be willing to pay to take an HIV test?
   1. I would only take an HIV test if it were free.
   2. I would pay up to 10 soles for an HIV test.
   3. I would pay up to 20 soles for an HIV test.
   4. I would pay up to 30 soles for an HIV test.
   5. I would not take an HIV test even if it were free.

**Treatment *to be asked for participants who are HIV positive only***

In the final section of this survey, we are going to ask you questions about specific features of how HIV treatment could be offered and which features are important to you when undergoing HIV treatment.

Location

Now we are going to ask you some hypothetical questions about HIV treatment services and your interest in different potential locations. Based on the following different locations that HIV treatment services that may be available, please indicate your level of interest given each hypothetical scenario.

If you were going to get HIV treatment, how important would it be for you to be able to get treatment at …

| **Location** | **Importance** | | | | |
| --- | --- | --- | --- | --- | --- |
| … a government clinic (EsSalud, MINSA)? | Extremely important | Somewhat important | Neither important nor unimportant | Somewhat unimportant | Extremely unimportant |
| … a CERITSS clinic? | Extremely important | Somewhat important | Neither important nor unimportant | Somewhat unimportant | Extremely unimportant |
| … a private clinic? | Extremely important | Somewhat important | Neither important nor unimportant | Somewhat unimportant | Extremely unimportant |
| … a non-governmental organization (Epicentro, Via Libre)? | Extremely important | Somewhat important | Neither important nor unimportant | Somewhat unimportant | Extremely unimportant |

Convenience

Now we are going to ask you some hypothetical questions about HIV treatment services and your interest in different factors that have to do with hours of availability and wait times. Based on the following different times that HIV treatment services may be available, please indicate your level of interest given each hypothetical scenario.

If you were going to receive treatment for HIV, how important would it be for you…

| **Situation** | **Importance** | | | | |
| --- | --- | --- | --- | --- | --- |
| … to be able to receive treatment on weekday evenings, after traditional working hours? | Extremely important | Somewhat important | Neither important nor unimportant | Somewhat unimportant | Extremely unimportant |
| … to be able to receive treatment on weekends? | Extremely important | Somewhat important | Neither important nor unimportant | Somewhat unimportant | Extremely unimportant |
| … to be able to receive treatment on weekday mornings? | Extremely important | Somewhat important | Neither important nor unimportant | Somewhat unimportant | Extremely unimportant |
| … to be able to receive treatment on weekday afternoons? | Extremely important | Somewhat important | Neither important nor unimportant | Somewhat unimportant | Extremely unimportant |
| … to not have to wait at the location to get treatment? | Extremely important | Somewhat important | Neither important nor unimportant | Somewhat unimportant | Extremely unimportant |
| … to schedule an appointment for your treatment? | Extremely important | Somewhat important | Neither important nor unimportant | Somewhat unimportant | Extremely unimportant |
| … that the location for getting treatment is close to your house? | Extremely important | Somewhat important | Neither important nor unimportant | Somewhat unimportant | Extremely unimportant |
| … that the location for treatment is in a neighborhood where you won’t run into anyone you know? | Extremely important | Somewhat important | Neither important nor unimportant | Somewhat unimportant | Extremely unimportant |
| … that the location for treatment is close to work? | Extremely important | Somewhat important | Neither important nor unimportant | Somewhat unimportant | Extremely unimportant |
| … that the location for treatment is easily accessible to public transportation? | Extremely important | Somewhat important | Neither important nor unimportant | Somewhat unimportant | Extremely unimportant |

Confidentiality/Privacy

Now we are going to ask you some hypothetical questions about HIV treatment services and your interest in different factors that have to do with confidentiality and privacy. Based on the following different situations, please indicate your level of interest given each hypothetical scenario.

How important is it to you that…

| **Situation** | **Importance** | | | | |
| --- | --- | --- | --- | --- | --- |
| …no assumptions be made regarding your HIV status? | Extremely important | Somewhat important | Neither important nor unimportant | Somewhat unimportant | Extremely unimportant |
| …no assumptions be made regarding your sexual identity because you have HIV? | Extremely important | Somewhat important | Neither important nor unimportant | Somewhat unimportant | Extremely unimportant |
| …you do not encounter anyone you know during your clinic visit? | Extremely important | Somewhat important | Neither important nor unimportant | Somewhat unimportant | Extremely unimportant |
| …your HIV status and other health information be kept private? | Extremely important | Somewhat important | Neither important nor unimportant | Somewhat unimportant | Extremely unimportant |
| …the place you receive HIV healthcare services is not known as a place where LGBTQ+ community goes for HIV care? | Extremely important | Somewhat important | Neither important nor unimportant | Somewhat unimportant | Extremely unimportant |

Cost

1. How much would you be willing to pay for each visit?
   1. I would only go for HIV care services if they were free.
   2. I would pay up to 25 soles for each clinic visit.
   3. I would pay up to 50 soles for each clinic visit.
   4. I would pay up to 75 soles for each clinic visit.
   5. I would not utilize HIV care services even if they were free.

Practices and preferences for HIV testing and treatment services amongst partners of transgender women in Lima, Peru: an exploratory, mixed methods study

Journal: PLoS One

Authors: Claudia Kazmirak, Deanna Tollefson*, Alexander Lankowski, Hugo Sanchez, Ivan Gonzales, Dianne Espinoza, Ann Duerr

*Corresponding author: [dtollefs@fredhutch.org](mailto:dtollefs@fredhutch.org) (Fred Hutchinson Cancer Center, Vaccine Infectious Disease Division)
