# Supplementary material for: Practices and preferences for HIV testing and treatment services amongst partners of transgender women in Lima, Peru: An exploratory, mixed methods study
Source: PLoS One. 2024 Jul 9;19(7):e0306852. doi: 10.1371/journal.pone.0306852 (PMC11232998; doi:10.1371/journal.pone.0306852)
Supplement: S1 Table — (DOCX) [file pone.0306852.s002.docx]

**Table S2. Satisfaction with HIV testing among transgender women (TW) and partners of transgender women (PTW) by facility**

|  | **EsSalud*** | | **MINSA**** | | **CERITS^#^** | | **Private clinic** | | **NGO** | | **Community Health Campaign** | |
| --- | --- | --- | --- | --- | --- | --- | --- | --- | --- | --- | --- | --- |
|  | **TW (n=7) n (%)** | **PTW (n=23) n (%)** | **TW (n=47) n (%)** | **PTW (n=122) n (%)** | **TW (n=48) n (%)** | **PTW (n=99) n (%)** | **TW (n=2) n (%)** | **PTW (n=21) n (%)** | **TW (n=16) n (%)** | **PTW (N=20) n (%)** | **TW (n=17) n (%)** | **PTW (n=8) n (%)** |
| **Satisfaction with services^&^** | | | | | | | | | | | | |
| Extremely satisfied | 4 (57.1) | 5 (21.7) | 13 (27.7) | 7 (5.7) | 14 (29.2) | 7 (7.1) | 2 (100) | 8 (38.1) | 6 (37.5) | 4 (20.0) | 4 (23.5) | 2 (25.0) |
| Somewhat satisfied | 2 (28.6) | 17 (73.9) | 28 (60) | 108 (88.5) | 27 (56.3) | 89 (89.9) | 0 | 12 (57.1) | 9 (56.3) | 15 (75.0) | 12 (70.6) | 4 (50.0) |
| Neither satisfied nor unsatisfied | 1 (14.3) | 0 | 3 (6.4) | 5 (4.1) | 5 (10.4) | 3 (3.0) | 0 | 1 (4.8) | 0 | 1 (5.0) | 0 | 1 (12.5) |
| Somewhat unsatisfied | 0 | 1 (4.3) | 3 (6.4) | 2 (1.6) | 1 (2.1) | 0 | 0 | 0 | 1 (6.3) | 0 | 0 | 0 |
| Extremely unsatisfied | 0 | 0 | 0 | 0 | 1 (2.1) | 0 | 0 | 0 | 0 | 0 | 1 (5.9) | 1 (12.5) |
| *Mean (SD)* | *1.6 (0.8)* | *1.9 (0.6)* | *1.9 (0.8)* | *2.0 (0.4)* | *1.9 (0.8)* | *2.0 (0.3)* | *1 (0)* | *1.7 (0.6)* | *1.8 (0.8)* | *1.9 (0.5)* | *1.9 (0.4)* | *2.3 (1.5)* |
| **Felt treated unfairly at this location^&^** | 1 (14.3) | 4 (17.4) | 7 (14.9) | 0 | 6 (12.5) | 1 (1.0) | 0 | 0 | 0 | 0 | 2 (11.8) | 0 |
| **Rationale – reasons for which participant felt they were treated unfairly^^^** | | | | | | | | | | | | |
| Because I am a transgender woman. | 0 | 0 | 7 (100) | 0 | 6 (100) | 0 | 0 | 0 | 0 | 0 | 2 (100) | 0 |
| Because I am a man who has sex with men. | 0 | 1 (25.0) | 4 (57.1) | 0 | 3 (50.0) | 0 | 0 | 0 | 0 | 0 | 1 (50.0) | 0 |
| Because I am gay. | 0 | 1 (25.0) | 6 (85.7) | 0 | 3 (50.0) | 0 | 0 | 0 | 0 | 0 | 1 (50.0) | 0 |
| Because I have sex with transgender women. | 0 | 1 (25.0) | 4 (57.1) | 0 | 4 (66.7) | 1 (100) | 0 | 0 | 0 | 0 | 1 (50.0) | 0 |
| Because I sell sex for money, goods and services. | 1 (100) | 0 | 3 (42.9) | 0 | 4 (66.7) | 0 | 0 | 0 | 0 | 0 | 1 (50.0) | 0 |
| Other | 0 | 1 (25.0) | 0 | 0 | 0 | 0 | 0 | 0 | 0 | 0 | 0 | 0 |
| Footnotes: *Government-run clinic for workers and their families, similar to social security system in the United States; **Government-run clinic administered by the Ministry of Health that serves as a safety net for the general population; ^#^Government-run STI clinic; ^&^Denominator is the ‘n’ that reported having received testing at the facility, which is available for each location/population in the column header. ^^^Denominator for each location/population is the ‘n’ presented in the “Felt treated unfairly at this location”. | | | | | | | | | | | | |

Accompanying paper: Practices and preferences for HIV testing and treatment services amongst partners of transgender women in Lima, Peru: an exploratory, mixed methods study

Journal: PLoS One

Authors: Claudia Kazmirak, Deanna Tollefson*, Alexander Lankowski, Hugo Sanchez, Ivan Gonzales, Dianne Espinoza, Ann Duerr

*Corresponding author: [dtollefs@fredhutch.org](mailto:dtollefs@fredhutch.org) (Fred Hutchinson Cancer Center, Vaccine Infectious Disease Division)
