# Supplementary material for: Practices and preferences for HIV testing and treatment services amongst partners of transgender women in Lima, Peru: An exploratory, mixed methods study
Source: PLoS One. 2024 Jul 9;19(7):e0306852. doi: 10.1371/journal.pone.0306852 (PMC11232998; doi:10.1371/journal.pone.0306852)
Supplement: S2 Table — (DOCX) [file pone.0306852.s003.docx]

**Table S3-1. Service preference means for HIV testing and treatment services, unadjusted for clustering***

|  | **Testing Services (HIV negative or unknown status)** | | | **Treatment Services (HIV positive)** | | |
| --- | --- | --- | --- | --- | --- | --- |
|  | TW** (n=43) mean (sd) | PTW^#^ (n=157) mean (sd) | p-value | TW (n=18) mean (sd) | PTW (n=6) mean (sd) | p-value |
| **Location^a^** | n=43 | n=157 |  | n=18 | n=6 |  |
| Government clinic^&^ | 1.28 (0.73) | 1.60 (0.84) | 0.01 | 1.33 (0.97) | 1.17 (0.41) | 0.7 |
| Government STI clinic^^^ | 1.26 (0.58) | 1.55 (0.77) | 0.01 | 1.17 (0.51) | 1.17 (0.41) | 0.5 |
| Private clinic | 1.42 (0.88) | 1.62 (0.89) | 0.09 | 1.33 (0.97) | 1.83 (1.60) | 0.2 |
| NGO (Epicentro, Via Libre) | 1.35 (0.84) | 1.60 (0.89) | 0.05 | 1.28 (0.57) | 1.83 (1.60) | 0.1 |
| Community health campaign | 1.26 (0.66) | 1.60 (0.83) | <0.01 | x | x | x |
| Mobile HIV testing van | 1.37 (0.87) | 1.66 (0.95) | 0.04 | x | x | x |
| Social venue (ex. Bar/club) or plaza (ex. San Martín) | 1.30 (0.71) | 1.80 (1.09) | <0.01 | x | x | x |
| “Self-test” for HIV that can be administered at home | 1.30 (0.64) | 1.72 (0.99) | <0.01 | x | x | x |
| Men’s health clinic | 1.28 (0.50) | 1.60 (0.85) | 0.01 | x | x | x |
| **Convenience^b^** |  |  |  |  |  |  |
| Access to testing or treatment on weekday evenings, after traditional working hours | 1.30 (0.71) | 1.55 (0.80) | 0.03 | 1.39 (0.98) | 1.17 (0.41) | 0.7 |
| Access to testing or treatment on weekends | 1.23 (0.48) | 1.52 (0.71) | <0.01 | 1.11 (0.32) | 1.17 (0.41) | 0.4 |
| Access to testing or treatment on weekday mornings | 1.33 (0.71) | 1.75 (1.03) | <0.01 | 1.22 (0.55) | 1.17 (0.41) | 0.6 |
| Access to testing or treatment on weekday afternoons | 1.37 (0.82) | 1.61 (0.83) | 0.05 | 1.11 (0.32) | 1.17 (0.41) | 0.4 |
| Wait ≤10 minutes at clinic for testing or treatment | 1.26 (0.58) | 1.55 (0.76) | 0.01 | 1.06 (0.24) | 1.17 (0.41) | 0.2 |
| Schedule appointment for testing or treatment | 1.19 (0.39) | 1.53 (0.74) | <0.01 | 1.11 (0.32) | 1.17 (0.41) | 0.4 |
| Access to testing or treatment at location close to home | 1.30 (0.77) | 1.55 (0.78) | 0.03 | 1.06 (0.24) | 1.17 (0.41) | 0.2 |
| Access to testing or treatment at location close to work | 1.30 (0.71) | 1.54 (0.76) | 0.03 | 1.06 (0.24) | 1.17 (0.41) | 0.2 |
| Access to testing or treatment at location easily accessible by public transit | 1.19 (0.39) | 1.58 (0.07) | <0.01 | 1.11 (0.32) | 1.17 (0.41) | 0.4 |
| **Confidentiality/Privacy^c^** |  |  |  |  |  |  |
| No one makes assumptions about HIV status^^^^ | 1.16 (0.37) | 1.52 (0.71) | <0.01 | 1.06 (0.24) | 1.83 (1.60) | 0.02 |
| No one makes assumptions about sexual identity^^^^ | 1.19 (0.39) | 1.54 (0.71) | <0.01 | 1.06 (0.24) | 1.17 (0.41) | 0.2 |
| You do not encounter anyone you know while testing | 1.35 (0.72) | 1.58 (0.76) | 0.04 | 1.17 (0.51) | 1.17 (0.41) | 0.5 |
| HIV status and health information are kept private | 1.21 (0.51) | 1.48 (0.69) | <0.01 | 1.06 (0.24) | 1.17 (0.41) | 0.2 |
| Test location not known as spot where LGBTQ+ go^^^^ | 1.26 (0.54) | 1.64 (0.87) | <0.01 | 1.06 (0.24) | 1.17 (0.41) | 0.2 |
| Footnotes: *The following Likert Scale was used for responses: 1 = extremely important, 2 = somewhat important, 3 = neither important nor unimportant, 4 = somewhat unimportant, 5 = extremely unimportant; **Transgender women; ^#^Partners of transgender women; ^a^Question stem read, “*If you were going to get an HIV test, how important would it be to test at ___”*; ^&^EsSalud/MINSA, state-sponsored healthcare networks for workers and their families (EsSalud) or for general population as a safety net administered by Ministry of Health (MINSA); ^^^CERITS clinics; ^b^Question stem read, *“If you were going to get an HIV test, how important would it be to __”*; ^c^Question stem read, *“If you were going to get an HIV test, how important would it be that ___”*; ^^When testing for HIV | | | | | | |

Accompanying paper: Practices and preferences for HIV testing and treatment services amongst partners of transgender women in Lima, Peru: an exploratory, mixed methods study

Journal: PLoS One Authors: Claudia Kazmirak, Deanna Tollefson*, Alexander Lankowski, Hugo Sanchez, Ivan Gonzales, Dianne Espinoza, Ann Duerr

*Corresponding author: [dtollefs@fredhutch.org](mailto:dtollefs@fredhutch.org) (Fred Hutchinson Cancer Center, Vaccine Infectious Disease Division)

**Table S3-2. Sensitivity Analysis – Service preference means for HIV testing and treatment services, adjusted for clustering***

|  | **Testing Services (HIV negative or unknown status)** | | | | **Treatment Services (HIV positive)** | | | | | |  |
| --- | --- | --- | --- | --- | --- | --- | --- | --- | --- | --- | --- |
|  | TW** (n=43) mean | PTW^#^ (n=157) mean | p-value | ICC | TW (n=18) mean | PTW (n=6) mean | p-value | | ICC | | |
| **Location^a^** | n=43 | n=157 |  |  | n=18 | n=6 |  | |  | | |
| Government clinic^&^ | 1.28 | 1.61 | <0.01 | 0.13 | 1.29 | 1.37 | 0.7 | | 0.74 | | |
| Government STI clinic^^^ | 1.26 | 1.55 | <0.01 | NA | 1.17 | 1.17 | 1 | | NA | | |
| Private clinic | 1.36 | 1.67 | 0.02 | 0.32 | 1.41 | 1.81 | 0.3 | | 0.88 | | |
| NGO (Epicentro, Via Libre) | 1.33 | 1.63 | 0.01 | 0.24 | 1.35 | 1.81 | 0.3 | | 0.80 | | |
| Community health campaign | 1.26 | 1.60 | <0.01 | NA | x | X | X | |  | | |
| Mobile HIV testing van | 1.35 | 1.67 | 0.02 | 0.21 | x | X | X | |  | | |
| Social venue (ex. Bar/club) or plaza (ex. San Martín) | 1.28 | 1.87 | <0.01 | 0.48 | x | X | X | |  | | |
| “Self-test” for HIV that can be administered at home | 1.28 | 1.75 | <0.01 | 0.17 | x | X | X | |  | | |
| Men’s health clinic | 1.27 | 1.61 | <0.01 | 0.12 | x | X | X | |  | | |
| **Convenience^b^** |  |  |  |  |  |  |  | |  | | |
| Access to testing or treatment on weekday evenings, after traditional working hours | 1.31 | 1.60 | 0.01 | 0.26 | 1.39 | 1.17 | 0.5 | | NA | | |
| Access to testing or treatment on weekends | 1.23 | 1.53 | <0.01 | 0.08 | 1.11 | 1.17 | 0.8 | | NA | | |
| Access to testing or treatment on weekday mornings | 1.32 | 1.82 | <0.01 | 0.32 | 1.22 | 1.17 | 0.8 | | NA | | |
| Access to testing or treatment on weekday afternoons | 1.36 | 1.63 | 0.05 | 0.15 | 1.11 | 1.17 | 0.8 | | NA | | |
| Wait ≤10 minutes at clinic for testing or treatment | 1.26 | 1.55 | 0.01 | NA | 1.06 | 1.17 | 0.5 | | NA | | |
| Schedule appointment for testing or treatment | 1.19 | 1.53 | <0.01 | NA | 1.11 | 1.17 | 0.8 | | NA | | |
| Access to testing or treatment at location close to home | 1.31 | 1.59 | 0.03 | 0.14 | 1.06 | 1.17 | 0.5 | | NA | | |
| Access to testing or treatment at location close to work | 1.30 | 1.55 | 0.04 | 0.06 | 1.06 | 1.17 | 0.5 | | NA | | |
| Access to testing or treatment at location easily accessible by public transit | 1.19 | 1.60 | <0.01 | 0.10 | 1.11 | 1.17 | 0.8 | | NA | | |
| **Confidentiality/Privacy^c^** |  |  |  |  |  |  |  | |  | | |
| No one makes assumptions about HIV status^^^^ | 1.16 | 1.52 | <0.01 | NA | 1.12 | 1.78 | 0.2 | | 0.62 | | |
| No one makes assumptions about sexual identity^^^^ | 1.19 | 1.54 | <0.01 | NA | 1.06 | 1.17 | 0.5 | | NA | | |
| You do not encounter anyone you know while testing | 1.33 | 1.59 | 0.03 | 0.16 | 1.17 | 1.17 | 1 | | NA | | |
| HIV status and health information are kept private | 1.21 | 1.48 | <0.01 | NA | 1.06 | 1.17 | 0.5 | | NA | | |
| Test location not known as spot where LGBTQ+ go^^^^ | 1.24 | 1.65 | <0.01 | 0.24 | 1.06 | 1.17 | 0.5 | | NA | | |
| Footnotes: *The following Likert Scale was used for responses: 1 = extremely important, 2 = somewhat important, 3 = neither important nor unimportant, 4 = somewhat unimportant, 5 = extremely unimportant; **Transgender women; ^#^Partners of transgender women; ^a^Question stem read, “*If you were going to get an HIV test, how important would it be to test at ___”*; ^&^EsSalud/MINSA, state-sponsored healthcare networks for workers and their families (EsSalud) or for general population as a safety net administered by Ministry of Health (MINSA); ^^^CERITS clinics; ^b^Question stem read, *“If you were going to get an HIV test, how important would it be to __”*; ^c^Question stem read, *“If you were going to get an HIV test, how important would it be that ___”*; ^^When testing for HIV | | | | | | | | |  | |  |
