# Supplementary material for: Practices and preferences for HIV testing and treatment services amongst partners of transgender women in Lima, Peru: An exploratory, mixed methods study
Source: PLoS One. 2024 Jul 9;19(7):e0306852. doi: 10.1371/journal.pone.0306852 (PMC11232998; doi:10.1371/journal.pone.0306852)
Supplement: S3 Table — (DOCX) [file pone.0306852.s004.docx]

**Table S4. Frequency and comparison of “extremely important” responses (TW vs PTW)**

|  | **HIV Testing** | | | | **HIV Treatment** | | | |
| --- | --- | --- | --- | --- | --- | --- | --- | --- |
|  | **TW^a^ (n=43)** n (%) | **PTW^b^ (n=157)** n (%) | chi-square | p-value | **TW (n=18)** n (%) | **PTW (n=6)** n (%) | chi-square | p-value |
| **Location^c^** |  |  |  |  |  |  |  |  |
| Government clinic^*^ | 35 (81.4) | 87 (55.4) | 9.6 | <0.01 | 15 (83.3) | 5 (83.3) | 0 | 1 |
| Government STI clinic^#^ | 34 (79.1) | 88 (56.1) | 7.5 | <0.01 | 16 (88.9) | 5 (83.3) | 0.13 | 0.7 |
| Private clinic | 32 (74.4) | 86 (54.8) | 5.4 | 0.02 | 15 (83.3) | 4 (66.7) | 0.76 | 0.4 |
| NGO (Epicentro, Via Libre) | 34 (79.1) | 89 (56.7) | 7.1 | <0.01 | 14 (77.8) | 4 (66.7) | 0.3 | 0.6 |
| Community health campaign | 36 (83.7) | 86 (54.8) | 11.9 | <0.01 | N/A | N/A | N/A | N/A |
| Mobile HIV testing van | 34 (79.1) | 84 (53.5) | 9.1 | <0.01 | N/A | N/A | N/A | N/A |
| Social venue (ex. Bar/club) or plaza (ex. San Martín) | 34 (79.1) | 77 (49.0) | 12.3 | <0.01 | N/A | N/A | N/A | N/A |
| “Self-test” for HIV that can be administered at home | 33 (76.7) | 80 (51.0) | 9.1 | <0.01 | N/A | N/A | N/A | N/A |
| Men’s health clinic | 32 (74.4) | 86 (54.8) | 5.4 | 0.02 | N/A | N/A | N/A | N/A |
| **Convenience^d^** |  |  |  |  |  |  |  |  |
| Test on weekday evenings, after traditional working hours | 33 (76.7) | 88 (56.1) | 6.1 | 0.01 | 15 (83.3) | 5 (83.3) | 0 | 1 |
| Test on weekends | 34 (79.1) | 88 (56.1) | 7.5 | <0.01 | 16 (88.9) | 5 (83.3) | 0.1 | 0.7 |
| Test on weekday mornings | 33 (76.7) | 80 (51.0) | 9.1 | <0.01 | 15 (83.3) | 5 (83.3) | 0 | 1 |
| Test on weekday afternoons | 32 (74.4) | 83 (52.9) | 6.4 | 0.01 | 16 (88.9) | 5 (83.3) | 0.1 | 0.7 |
| Wait ≤10 minutes to test | 34 (79.1) | 87 (55.4) | 7.9 | <0.01 | 17 (94.4) | 5 (83.3) | 0.7 | 0.4 |
| Schedule appointment for test | 35 (81.4) | 87 (55.4) | 9.6 | <0.01 | 16 (88.9) | 5 (83.3) | 0.1 | 0.7 |
| Test at location close to home | 35 (81.4) | 87 (55.4) | 9.6 | <0.01 | 17 (94.4) | 5 (83.3) | 0.7 | 0.4 |
| Test at location close to work | 33 (76.7) | 87 (55.4) | 6.4 | 0.01 | 17 (94.4) | 5 (83.3) | 0.7 | 0.4 |
| Test at location easily accessible by public transit | 35 (81.4) | 88 (56.1) | 9.2 | <0.01 | 16 (88.9) | 5 (83.3) | 0.1 | 0.7 |
| **Confidentiality/Privacy^e^** |  |  |  |  |  |  |  |  |
| No one makes assumptions about HIV status^^^ | 36 (83.7) | 88 (56.1) | 11.0 | <0.01 | 17 (94.4) | 4 (66.7) | 3.1 | 0.08 |
| No one makes assumptions about sexual identity^^^ | 35 (81.4) | 86 (54.8) | 10.0 | <0.01 | 17 (94.4) | 5 (83.3) | 0.7 | 0.4 |
| You do not encounter anyone you know while testing | 33 (76.7) | 84 (53.5) | 7.5 | <0.01 | 16 (88.9) | 5 (83.3) | 0.1 | 0.7 |
| HIV status and health information are kept private | 36 (83.7) | 92 (58.6) | 9.3 | <0.01 | 17 (94.4) | 5 (83.3) | 0.7 | 0.4 |
| Test location not known as spot where LGBTQ+ go^^^ | 34 (79.1) | 84 (53.5) | 9.1 | <0.01 | 17 (94.4) | 5 (83.3) | 0.7 | 0.4 |
| Footnotes: ^a^Transgender women; ^b^Partners of transgender women; ^c^Question stem read, “*If you were going to to get an HIV test, how important would it be to test at ___”*; *EsSalud/MINSA, state-sponsored healthcare networks for workers and their families (EsSalud) or for general population as a safety net administered by Ministry of Health (MINSA); ^#^CERITSS; ^d^Question stem read, “*If you were going to to get an HIV test, how important would it be to ___”*; ^e^Question stem read, “*If you were going to to get an HIV test, how important would it be that ___”*; ^^^When testing for HIV | | | | | | | | |

Accompanying paper: Practices and preferences for HIV testing and treatment services amongst partners of transgender women in Lima, Peru: an exploratory, mixed methods study

Journal: PLoS One Authors: Claudia Kazmirak, Deanna Tollefson*, Alexander Lankowski, Hugo Sanchez, Ivan Gonzales, Dianne Espinoza, Ann Duerr

*Corresponding author: [dtollefs@fredhutch.org](mailto:dtollefs@fredhutch.org) (Fred Hutchinson Cancer Center, Vaccine Infectious Disease Division)
