# Supplementary material for: Practices and preferences for HIV testing and treatment services amongst partners of transgender women in Lima, Peru: An exploratory, mixed methods study
Source: PLoS One. 2024 Jul 9;19(7):e0306852. doi: 10.1371/journal.pone.0306852 (PMC11232998; doi:10.1371/journal.pone.0306852)
Supplement: S4 Table — (DOCX) [file pone.0306852.s005.docx]

**Table S5. Service preference means for HIV testing services by PTW type^x^**

|  | **Total PTW* (n= 157)** | **PTW subtype** | | | **One-way ANOVA**  p-value |
| --- | --- | --- | --- | --- | --- |
|  | mean (sd) | **Stable (n=12)**  mean (sd) | **Casual (n=78)**  mean (sd) | **Transactional (n=67)**  mean (sd) |  |
| **Location^&^** |  |  |  |  |  |
| Government clinic** | 1.60 (0.84) | 1.75 (1.36) | 1.73 (0.88) | 1.42 (0.63) | 0.07 |
| Government STI clinic^#^ | 1.55 (0.77) | 1.75 (1.29) | 1.63 (0.77) | 1.43 (0.63) | 0.2 |
| Private clinic | 1.62 (0.89) | 2.00 (1.54) | 1.63 (0.77) | 1.43 (0.63) | 0.08 |
| NGO (Epicentro, Via Libre) | 1.60 (0.89) | 2.33 (1.72) | 1.62 (0.76) | 1.45 (0.76) | <0.01 |
| Community health campaign | 1.60 (0.83) | 2.25 (1.60) | 1.67 (0.80) | 1.40 (0.58) | <0.01 |
| Mobile HIV testing van | 1.66 (0.95) | 2.33 (1.56) | 1.72 (0.91) | 1.46 (0.81) | <0.01 |
| Social venue (ex. Bar/club) or plaza (ex. San Martín) | 1.80 (1.09) | 2.50 (1.51) | 1.92 (1.13) | 1.54 (0.88) | <0.01 |
| “Self-test” for HIV that can be administered at home | 1.72 (0.99) | 2.17 (1.40) | 1.86 (1.07) | 1.48 (0.75) | 0.02 |
| Men’s health clinic | 1.60 (0.85) | 2.00 (1.41) | 1.63 (0.76) | 1.49 (0.80) | 0.1 |
| **Convenience^a^** |  |  |  |  |  |
| Test on weekday evenings, after traditional working hours | 1.55 (0.80) | 1.75 (1.29) | 1.69 (0.90) | 1.36 (0.48) | 0.03 |
| Test on weekends | 1.52 (0.71) | 1.42 (0.67) | 1.63 (0.81) | 1.40 (0.58) | 0.2 |
| Test on weekday mornings | 1.75 (1.03) | 3.17 (1.59) | 1.71 (0.87) | 1.54 (0.89) | <0.01 |
| Test on weekday afternoons | 1.61 (0.83) | 2.17 (1.40) | 1.67 (0.85) | 1.43 (0.61) | 0.01 |
| Wait ≤10 minutes to test | 1.55 (0.76) | 2.08 (1.24) | 1.60 (0.81) | 1.39 (0.52) | <0.01 |
| Schedule appointment for test | 1.53 (0.74) | 1.75 (1.22) | 1.62 (0.79) | 1.39 (0.52) | 0.1 |
| Test at location close to home | 1.55 (0.78) | 1.83 (1.34) | 1.62 (0.79) | 1.42 (0.61) | 0.1 |
| Test at location close to work | 1.54 (0.76) | 2.00 (1.54) | 1.60 (0.73) | 1.39 (0.52) | 0.02 |
| Test at location easily accessible by public transit | 1.58 (0.07) | 2.25 (1.71) | 1.62 (0.84) | 1.42 (0.61) | <0.01 |
| **Confidentiality/Privacy^b^** |  |  |  |  |  |
| No one makes assumptions about HIV status^^^ | 1.52 (0.71) | 1.75 (1.06) | 1.60 (0.80) | 1.39 (0.49) | 0.1 |
| No one makes assumptions about sexual identity^^^ | 1.54 (0.71) | 1.83 (1.03) | 1.62 (0.79) | 1.39 (0.49) | 0.05 |
| You do not encounter anyone you know while testing | 1.58 (0.76) | 1.92 (1.24) | 1.68 (0.81) | 1.40 (0.52) | 0.03 |
| HIV status and health information are kept private | 1.48 (0.69) | 1.83 (1.27) | 1.55 (0.70) | 1.34 (0.48) | 0.03 |
| Test location not known as spot where LGBTQ+ go | 1.64 (0.87) | 1.67 (1.23) | 1.78 (0.92) | 1.46 (0.70) | 0.09 |
| Footnotes: : ^x^The following Likert Scale was used for responses: 1 = extremely important, 2 = somewhat important, 3 = neither important nor unimportant, 4 = somewhat unimportant, 5 = extremely unimportant; *Partners of transgender women; ^&^Question stem read, “*If you were going to to get an HIV test, how important would it be to test at ___”*; **EsSalud/MINSA, state-sponsored healthcare networks for workers and their families (EsSalud) or for general population as a safety net administered by Ministry of Health (MINSA); ^#^CERITS; ^a^Question stem read, *“If you were going to get an HIV test, how important would it be to __”*; ^b^Question stem read, *“If you were going to get an HIV test, how important would it be that ___”*; ^When testing for HIV | | | | | |

Accompanying paper: Practices and preferences for HIV testing and treatment services amongst partners of transgender women in Lima, Peru: an exploratory, mixed methods study

Journal: PLoS One

Authors: Claudia Kazmirak, Deanna Tollefson*, Alexander Lankowski, Hugo Sanchez, Ivan Gonzales, Dianne Espinoza, Ann Duerr

*Corresponding author: [dtollefs@fredhutch.org](mailto:dtollefs@fredhutch.org) (Fred Hutchinson Cancer Center, Vaccine Infectious Disease Division)
