# Supplementary material for: Practices and preferences for HIV testing and treatment services amongst partners of transgender women in Lima, Peru: An exploratory, mixed methods study
Source: PLoS One. 2024 Jul 9;19(7):e0306852. doi: 10.1371/journal.pone.0306852 (PMC11232998; doi:10.1371/journal.pone.0306852)
Supplement: S5 Table — (DOCX) [file pone.0306852.s006.docx]

**Table S6. Themes regarding service preferences from qualitative interviews**

| **Theme** | **Explanation** | **Quotes** |
| --- | --- | --- |
| **Need for more information about PrEP/HIV testing services in general** | - Need for more awareness - Also explain clearly so that people know they still need to protect themselves in other ways - Information not currently reaching populations - Excitedness and initiative to share information with friends | "Sure, it's a good option. But it's very… it is a topic very much like a myth, because it is not exposed to people as it should be…. This subject is spoken of more as a taboo, as something closed." (TW #1)  "If you explain it to me, just as you are explaining to me openly to an open audience right now, I think the interest would be greater." (TW #1)  "The advantage would be that it helps you prevent [HIV] and the disadvantage would be that, if you do not give people good information, they may think that if they use PrEP, it is no longer necessary to use a condom, and they can get infected." (PTW #13 – stable)  "P: You're telling me it's something that's going to happen?  E: No, it already exists" (PTW #14 – stable)  "I am listening it can be a little scary because it can be an experiment and they are just trying, but if you tell me that it already exists in several countries, in that case I could try it, but with someone's guarantee that it is not an experiment and that I will not have any side effects." (PTW #14 – stable)  "I haven't found any campaigns or places where I could take it. But if I find one, of course I will sign up for the treatment." (PTW #7 – stable)  "If I had known that five years ago, I would have been all in. If possible, the rest of my life would have continued with that. But, if nothing else, I believe it is a good thing that these pills are now available. You know what? I'm going to recommend that to my friends because a lot of my friends, when they lose their minds, [they] just have bareback sex and that's it." (PTW #1 – casual)  ****in regards to improving HIV services:***  “I think… with information, more information. You should get more information and this needs to get regularized, the taboo needs to go away. I think and know many friends and acquaintances that suspect they already have it but don’t go to take the test or don’t want to get treatment because they’re scared about what others are going to say, the comments, that society is going to judge and separate them. I think that’s because of the lack of knowledge and ignorance, right?” (PTW #5 – transactional)  “E: And what do you think could make people more interested in PrEP?  P: The information. Well, the good information because… See, just about an hour and thirty minutes before you called me, I was talking with a friend that told me that he was taking PrEP and because of that, he felt like he didn’t have a risk when not using condoms. And I was explaining to him that, as far as I’m concerned, it’s not like that, right? I mean, just because he’s taking PrEP, that doesn’t mean that he doesn’t have to take care.” (PTW #5 – transactional) |

| **Theme** | **Explanation** | **Quotes** |
| --- | --- | --- |
| **Discrimination in healthcare settings** | - Calling TW by their “male” name - Discrimination observed by both TW and PTW, but especially of TW - Some discrimination of PTW noted | "P: There is discrimination, but impulsive. It is not because they want to, what happens is that in the CERITS they treat you by your social name, but unfortunately in “Seguro” there is no intention, they treat you as you are  E: Ah, they call you, for example, the name of the DNI (identity document), the name of a man." (TW #4)  "P: Not with me, but with my partner in 2020, we went to do the test and something happened that bothered me, but not for me but for my partner who called my trans partner with his male name because and that bothered me  E: Sure.  P: To give us the result  E: To give them their result, they called him by his man's name. Okay. Did you do anything? Did they do anything? Did they complain or not?  P: Well, she didn't come over. As they called by man's name I approached and in a low voice I told the young lady to quietly call her by her last name or to tell her her name Kassandra, but her name is not Kassandra, then call her by her last name because she is a trans woman and she told me okay and called her by her last name." (PTW #14 – stable)  "I think it's not like that for the girls… because I've seen that they keep them waiting, don't attend to them fast enough, and don’t make things easier for them…. I mean, like they exclude them. They minimize them." (PTW #3 – stable)  "P: Of course, because before it was very ugly. Because sometimes they did it to laugh at you because sometimes they saw that you were a trans girl, they called you by the name that is on your ID. And you know there are people in the hospital; there are boys and ladies; everyone turns to look at you with your name; it's a shame for us." (TW #2)  “At the time of the test, I had the impression that the [clinic worker] was disgusted by me… I asked him why he was discriminating against me like that, and if he had a problem with me, he should have told me. Because it shouldn’t be like that… He replied to me in a bad way, but I just left it like that. But I did tell the manager about it. Because one guy from there asked me, and I told him that I was never going back because the treatment wasn’t right.” (PTW #8 – casual)  “E: Okay, and when they are straight you feel that they make some comments?  P: More than comments, it’s the way that they attend to you or the gestures they do when you mention certain stuff, I don’t know, you feel like you’re being prejudged.” (PTW #5 – transactional)  "They look at you kind of weird. The nurses, they are surprised, they are not like other places I have gone where they treat you normally." (PTW #13 – stable) |

| **Theme** | **Explanation** | **Quotes** |
| --- | --- | --- |
| **Importance of interpersonal support in HIV testing** | - Between HIV positive people - Support within the healthcare system - Promoters who escort patients to get tested/treated - Peer-to-peer support counselors | "When my partner said a gang, a group of people with all positive, got together on weekends or the end of the month"…."and we told our experience, the comments of each one, that he was patient" (TW #3)  "The promoters helped me, picked me up, took me and helped me with my stay there or did the paperwork and it was fast" (TW #4)  "P: I like that when you get there, there are some people outside who are like… how can I say this? As an example, there are promoters who will greet you, bring you more information about what you want to do, and assist you.  E: So, there are people that support you; we could say there are peer-to-peer counselors or something like that." (TW #2)  "The [patients/clinic staff] you get to meet, the friends you make. That's the best part. And the assurance they provide." (PTW #7 – stable)  "First and foremost, they explained the subject and everything, that I should not be embarrassed, that I should lose all signs of shyness, and that I should feel good, and that I should feel good, and that it was not something from another world. And nothing, I felt great, and they were very nice to me.” (PTW #1 – casual) |
| **Need for increased efficiency of HIV testing and treatment services** | - Timing; having to wait long and taking a very long time for appointment - Needing appointment but having difficulty making one | "I think that they should do campaigns, but like in some groups, like, let's say, groups of 20 people, so that it doesn't get agglomerated and also doesn't get messy. And like that, it would get faster. And also, there should be a room for counseling, a room for the blood tests, and a room for the doctors, so it would be fast, people would go to the rooms as fast as possible." (PTW #7 – stable)  "The tests are quick, but the waiting is the issue." (PTW #7 – stable)  "That you have to wait hours, after hours" (PTW #1 – casual)  "You would have to be one of the first, leave the house a little earlier or maybe get up early to be one of the first within the number of people they are asking for." (PTW #12 – transactional)  “Some time ago, I went to a public establishment and they said they couldn’t attend me because they didn’t have the things, the tests or haven’t received them.” (PTW #5 – transactional)  “Generally, those are fast tests, they say: ‘’40 minutes and the results are out’’ But sometimes I think they exceed the capacity and there’s too many people so you get to wait 2 and a half hours for the results and that’s not the time they said.” (PTW #5 – transactional) |

| **Theme** | **Explanation** | **Quotes** |
| --- | --- | --- |
| **Importance of LGBTQ+ representation amongst providers** | - Gay/trans women as healthcare workers/administering test | "The thing is that, generally, the places that you definitely go to for STDs or HIV, are run by gays. When they’re gays, it' s more comfortable because they understand you. But, sometimes I’ve had attention from straight people and they always, somehow, make some gestures or comments that aren’t right." (PTW #5 – transactional) |
| **Need for greater focus on confidentiality, privacy and discretion in HIV testing services** | - Sharing of HIV status - Asking sensitive questions to multiple patients in the same room - Mobile testing vans being not very discrete | “E: Let's say the most complicated thing. What do you think can be done to improve HIV testing services? What would you suggest?  P: For HIV services confidentiality  E: That they improve confidentiality, did you have any problems regarding that?  P: No, but there are other people who do.  E: You've seen other people who do. How which cases? Did anyone count their results? Were they exposed? P: Sure.  E: Did they count the results of others? Who counted the results of others? What kind of staff?  P: The same counselor or second people, but that is well known to be punishable.  E: Okay, but where were they telling it or in what situation were they telling the result of others?  P: It can be through social media  E: Did you place positive results on social networks?  P: Sure, in groups and all that.  E: And they didn't denounce that?  P: They didn't denounce, they just stayed silent or disappeared from the map and that's it.” (PTW #12 – transactional)  “E: Is there something that would make you feel more comfortable when talking about your sex life? Any elements?  P: Well, mostly I think that being confidential, it has happened to me, many times, that there are other people in there. Or sometimes, because of the time, they attend two people and make the questions right after the other.  E: Ah, okay. You would say that taking care about confidentiality and privacy.” (PTW #5 – transactional)  “E: And what do you dislike the most? P: The gossip.  E: Have you seen some cases? Or has it happened to you?  P: Yes, yes, yes, at least once, and it was very annoying to me. I even had to call them out… There was a guy leaving and it was my turn after him, so I got in there and there was the nurse with the doctor and they didn’t even care that I was there, they started saying ‘’Oh, the boy that left, he was positive’’ and stuff like that. I called them out saying that it wasn’t proper for me that they were making those comments and it was worse that someone that had nothing to do with it was there, right? Because I didn’t have the right to know if the guy was positive or not.” (PTW #5 – transactional)  “There are things we don't like people to find out. As there in that square there are many comments from everyone. That's why I went to Patrucco… Because one enters that mobile [testing van]. You know that this motive is for those results." (PTW #11 – transactional) |

Accompanying paper: Practices and preferences for HIV testing and treatment services amongst partners of transgender women in Lima, Peru: an exploratory, mixed methods study

Journal: PLoS One

Authors: Claudia Kazmirak, Deanna Tollefson*, Alexander Lankowski, Hugo Sanchez, Ivan Gonzales, Dianne Espinoza, Ann Duerr

*Corresponding author: [dtollefs@fredhutch.org](mailto:dtollefs@fredhutch.org) (Fred Hutchinson Cancer Center, Vaccine Infectious Disease Division)
