# Supplementary material for: Practices and preferences for HIV testing and treatment services amongst partners of transgender women in Lima, Peru: An exploratory, mixed methods study
Source: PLoS One. 2024 Jul 9;19(7):e0306852. doi: 10.1371/journal.pone.0306852 (PMC11232998; doi:10.1371/journal.pone.0306852)
Supplement: Attachment — Submitted filename: KazmirakTollefson_ResponseToReviewers_Feb9_2024.docx [file pone.0306852.s007.docx]

**Response to Reviewers**

**Manuscript:** Practices and preferences for HIV testing and treatment services amongst partners of transgender women in Lima, Peru: an exploratory, mixed methods study

**Reviewer #1**: Thank you for the opportunity to review, “Practices and preferences for HIV testing and treatment services amongst partners of transgender women in Lima, Peru: an exploratory, mixed methods study.” The authors present results of a mixed-methods survey of transwomen (TW) and their sexual partners (PTW) in Lima, Peru regarding their preferences and practices for HIV testing and treatment. The results are interesting and important, presenting a relatively large sample of PTW (a population that has been understudied to date) addressing differences in attitudes towards and use of HIV prevention and testing services. The analysis is well done and the manuscript well written. There are a few points for clarification or elaboration prior to publication:

1) **The authors describe their sampling method as Respondent Driven Sampling (RDS) but do not use any of the analytic methods inherent to RDS, and the single-wave of recruitment from seeds selected through convenience sampling is not consistent with RDS methods. It may be preferable to describe recruitment as peer recruitment or snowball sampling to avoid methodological disputes over terminology.**

*Response:* Thank you for pointing out this difference. We agree that this study utilized a simplified form of RDS due to using only one wave of recruitment. However, we feel it is appropriate to use of the term “Respondent Driven Sampling” because we retained the dual incentive structure that is used with RDS, despite adopting only one wave of sampling. We believe this keeps our approach in line with the methodology as outlined in the seminal RDS paper by Heckathorn: DD Heckathorn “Respondent-Driven Sampling: A new approach to the study of hidden populations.” Social Problems. 1997. 44(2): 174-19. (See: <https://doi.org/10.2307/3096941>)

We have clarified use of this terminology in the manuscript. Please see lines 103-104.

**2) In the abstract, it would help to specify the numbers of TW and PTW recruited for the study.**

*Response:* Thank you for this suggestion. We have now added the specific numbers of PTWs and TWs that were included in the survey and interview in the abstract (see lines 10-11). In the ‘Methods’ section of the abstract, it now reads:

*“Using an explanatory sequential design, we administered online surveys to PTW (n=165) and TW (n=69), then interviewed a subset of participants (n=20: 16 PTW, 4 TW).”*

**3) The Background is brief and does not provide any context for understanding why the authors chose to address differences among PTW according to their partnership status or partner type with TW. Given that Jess Long’s papers, cited in the manuscript, provide a great amount of detail on this topic, it would help to have some discussion of this work in the Background to set the stage for the rest of the analysis.**

*Response:* Thank you for noting this. We have substantially expanded upon the second paragraph of the introduction, with specific reference to Jess Long’s work. We believe the additions made to the introduction continue to keep the introduction succinct but clarify how this study expands on the work done by Jess Long and her colleagues. Additions to this paragraph that specifically highlight Jess Long’s work are noted in red text below.

*In Peru, roughly 30% of TW are estimated to be living with HIV, with high levels of HIV transmission occurring within TW and PTW sexual networks (14,15). Extensive research by Long et al. has demonstrated that PTW in Peru are distinct from MSM and that research is needed to understand how to best reach this population with HIV prevention and treatment services (6–9,13). The invisibility and heterogeneity of PTW has made it difficult to assess their knowledge of HIV, self-perceived HIV risk, and history of, or preferences for, HIV testing services (6,7,9,11). This is problematic, as such information is key to developing effective programs tailored to the needs of this historically underserved population.* *Traditional HIV testing, prevention, and treatment services in Peru are offered to anyone through the national health system at CERITS (government-run STI clinics) and there has been substantial outreach to reach TW through non-traditional services (community-based or venue-based testing) (16,17). However, neither traditional nor non-traditional HIV services have specifically targeted PTW, thus past research needs to be expanded to understand how to best reach this population with HIV prevention and care services (6–9,13). In addition, the heterogeneity of PTW in Peru, as highlighted by past research (6,7), highlights the additional need to explore how such services should be tailored to reach PTW based on the type of relationship they have with TW.* *The need to elucidate how to best reach PTW is especially urgent, as the inclusion of PrEP to Peru’s national HIV prevention guidelines in June 2023 ushers in opportunities to offer HIV prevention services to both TW and PTW (18), which are overdue.*

**4) In the Methods, participants are required to have been “literate in Spanish.” Does this comment mean that participants who were unable to read or write were excluded from participation?**

*Response:* Yes, this means people who were unable to read and write in Spanish would be excluded from participation in this study. However, to the best of our knowledge, there were no individuals turned away from this study due to illiteracy in Spanish, thus we do not believe this criteria affected participation. Peru has very high literacy rates (with some estimating it to be greater than 99%) (Source: [Global Data](https://www.globaldata.com/data-insights/macroeconomic/literacy-rate-in-peru/#:~:text=The%20literacy%20rate%20reached%2099.26,increased%20by%200.1%25%20in%202021.&text=Literacy%20is%20defined%20by%20the,of%20a%20particular%20age%20group.)); even if literacy amongst TW is lower than the general population, we did not encounter illiteracy amongst TW or PTW to be a barrier to this study, nor have we experienced challenges with illiteracy in this population when conducting other studies in Peru.

**5) What were the recruitment patterns for TW seeds and the PTWs (i.e., were a few seeds responsible for a great majority of the PTWs, what was the distribution of PTW characteristics in relation to TW seeds, etc.)?**

*Response*: This is an excellent question. We have clarified in our manuscript that each TW could recruit up to 5 people (see line 101). The table below reveals how many TW recruited 0, 1, 2, 3, 4, or 5 PTWs for the study. Typically, TWs recruited 2-3 PTW to participate in this study (average: 2.4), although 15 TW did not recruit any PTW and 12 each recruited 5 PTW. We have added language to address this in the first paragraph of the results section (see lines 181-182).

| **No. PTW Recruits per TW** | **No. TW** | **Total No. PTW in study** |
| --- | --- | --- |
| 0 | 15 | 0 |
| 1 | 8 | 8 |
| 2 | 14 | 28 |
| 3 | 11 | 33 |
| 4 | 9 | 36 |
| 5 | 12 | 60 |
| **Total** | **69** | **165** |

Regarding the distribution of PTW characteristics by TW seed, we reviewed data for education, income, and job status and found no noteworthy trends to report; we generally saw that there was much heterogeneity amongst partners for each TW. (There were too few participants per seed to make meaningful comparisons of age.) When there were multiple PTW per TW seed, there was wide variation in education level, job status, and income for each TW seed. This is captured in the figures below.

6) What were the **demographic and other characteristics of the subset of participants in the qualitative interviews**?

*Response:*  Thank you for this question. In our manuscript, we provide an overview of who was interviewed in lines 190-193. This information was gathered during the interviews. The participant’s interview was not connected to their survey, so we are not able to abstract more detailed demographic data on the interviewees at this time. However, we believe the following information, as presented in our manuscript provides sufficient data on the interviewees:

*“We conducted follow-up interviews with 20 survey participants: four with TW and 16 with PTW. Of PTW interviewees, 5 were stable partners, 6 were casual partners, and 5 were transactional partners of the referring TW. All TW interviewees identified as sex workers, compared with one-quarter (n=4) of PTW. Three-quarters of TW interviewees were living with HIV, compared to one-quarter of PTW interviewees.”*

**7) Please specify in the Methods how “Stable partners” were defined in the survey (similar to the explanations provided for casual and transactional partners).**

*Response:* Thank you for this question. We have included the definition for “stable partners” in the methods section (see lines 139-140): “*PTW were classified as stable partners if they self-reported the referring TW to be their ‘stable partner/spouse’.”* The definition for stable partner was derived directly from Answer “B” in Question 10 of the survey, which is included in the Appendix. The question was asked as follows:

*Question 10: What is/was your relationship with the transgender woman who referred you? *Only to be asked for persons who indicated sex assigned at birth was “male” and current gender identity is “man.”*

a) She is a friend/not a sexual partner
b) She is a stable partner/spouse
c) She is a casual partner/friends-with-benefits
d) She is a punto/one-time partner (we had sex once and did not exchange money, goods or services in exchange for the sex)
e) She is a client partner (I sold sex to her)
f) She is a partner from whom I purchased sex (she sold sex to me)

**8) Was the qualitative data analyzed in the original Spanish or in English translation?**

*Response*: The qualitative data were analyzed using transcripts which had been translated into English, as the qualitative analysis was conducted by native English speakers. However, a native Spanish speaker who had conducted the interviews and was very familiar with the Spanish data, reviewed the qualitative analysis and was in agreement with the findings. When questions about the qualitative data and its meaning arose, the original Spanish transcripts were consulted.

We clarified this in the methods section. Please see lines: 163-168.

*“After analyzing survey data, we conducted a rapid thematic analysis of the interview data* ***in English*** *using the framework method [19,20]. Two analysts (CK, DT) independently reviewed all transcripts, then met to discuss any differences in their reviews, come to consensus, and identify key themes that emerged from the data;* ***Spanish-language transcripts were consulted if questions arose over meaning of the text.****”*

**9) Why was Via Libre’s IRB used for this study (a brief explanation would be useful)?**

*Response:* Thank you for this question. The Peruvian organization that was conducting this study (EpiCentro) does not have its own IRB; as a result, it relies on the IRB at Via Libre, another research organization in Lima, to review its protocols. The Via Libre IRB is well established and respected in the research community. It is a known IRB that welcomes applications from organizations across Peru and we have used it for other studies in the past.

We have clarified this in the manuscript. Please see lines 171-173. For convenience, we have pasted this section below.

*“The study was approved by ethics boards at Via Libre (Lima, Peru) and University of Washington (Seattle, USA). Via Libre is a non-governmental organization dedicated to conducting research and providing services for people affected by HIV in Peru; we have used its IRB frequently for local review of HIV-related studies amongst sexual minorities (e.g., 23).”*

**10) The right side of Table 2 is cut off in the current formatting.**

*Response:* Thank you for noting that Table 2 is cut off using the current formatting. We have formatted the paper in this manner to follow what we understand to be PLoS One’s guidelines for submission. The full table can be seen under “Draft” view. We have included a snapshot of the table below for your convenience.

**11) In the Discussion, the authors suggest that power differentials in the relationships between TW and PTW are partly due to the higher SES of PTW. However, in this sample, over 95% of all participants (and 94% of PTW) report earning less than $400 USD/month. It is more likely that other factors, like gendered power differentials between cis-men and transwomen, and the high frequency of sex workers in the TW sample, play a greater role in defining power differentials in these relationships.**

*Response:* Thank you for raising this point. We agree that other factors, like gendered power differentials between cis and transwomen, likely play a great role in the power differentials in these relationships. This is a common observation across cultures and is likely to be true in the Peruvian setting as well. However, we also found in our study that there appeared to be substantial differences in socioeconomic status between the TW and PTW in this study. These differences are viewed in Table 1.

- Level of Education: 27% of PTW report having a post-secondary education, compared to only 7% of TW.
- Employment Status: 46% of PTW report have a full time job, in comparison to 6% of TW.
- Income level: Almost 34% of PTW had an income in the prior month of more than 1,000 sole in comparison to only 4% of TW. The national minimum monthly wage in Peru at the time of this study was 930 sole/month ([Peru - Minimum wages 2022 | countryeconomy.com](https://countryeconomy.com/national-minimum-wage/peru)); while this is very low, it does suggest that vastly more PTW were earning more than minimum wage in the month prior to this study, in comparison to PTW.

We cannot state that it was the difference in SES or gender (or some combination of both) that contributed to power differentials observed in our study. As such, we have amended the language in the discussion section to broadly describe these factors could have contributed to the power differential we observed. This discussion now reads as follows (see lines 324-331):

*Other slight differences noted between TW and PTW may be due to the power differential between these populations. Power differences between TW and their partners have been well-noted in the literature, largely due to cis males being in the greatest position of power in many societies (1,3,7,10); our study also observed PTW to be of higher socioeconomic status than TW, as evidenced by PTW generally having higher levels of education, employment, and income than their TW partners, which may further increase the power differential between PTW and TW. Because PTWs have more power in society than TWs, we hypothesize that PTWs may be able to be more flexible in accessing HIV services and thus feel less strongly than TW about how, when, or where services are provided.*

**12) The authors seem to gloss over a potentially fascinating observation in their qualitative data, where PTW complained about on-site HIV testing with vans or mobile units as these can increase visibility of individuals waiting outside the van for testing, and some stating that they prefer to attend traditional testing venues as they provide greater privacy and discretion, while the TW interviewed did not have similar complaints. This observation could have critical implications for how HIV testing and treatment is provided to these different groups, and merits greater discussion.**

*Response:* Thank you for this note and for such careful reading of our results. We agree this is a fascinating finding, and it was underemphasized in our original submission. We also want to emphasize that this finding is based on a small number of interviews, so it should be interpreted cautiously. That said, we agree it should be further emphasized in the discussion of this paper. We have rearranged the discussion section and added a few sentences to the third paragraph to address this (Lines 345-351). The addition is bolded in the excerpt below:

*Ultimately, the qualitative interviews in this study were most useful in highlighting how HIV services could be tailored to better reach PTW in Lima. The overarching principles important to PTW also rang true for TW, suggesting a focus on these core elements could improve reach to both PTW and TW: implementing procedures to reduce wait times for testing and results and strengthening sensitization for healthcare workers on patient privacy and transgender care.* ***However, interviews also highlighted a core difference in how PTW may wish to interact with HIV services, in comparison to TW. PTW, specifically those not in stable relationships with TW, voiced concern over the lack of privacy offered by some non-traditional testing events, namely on-site, mobile HIV testing units; such concerns were not voiced by TW in our study. While this finding is based on a small number of interviews, it echoes findings in past qualitative research (6), suggesting that while PTW and TW have similar preferences for many aspects of HIV testing, PTW, specifically those not in stable relationships with TW, may be better reached through traditional testing venues.***

**Reviewer #2:** In article ‘Practices and preferences for HIV testing and treatment services amongst partners of transgender women in Lima, Peru: an exploratory, mixed methods study’ submitted for review at PLOS One, the authors conducted a cross-sectional mixed methods study among Partners of Transgender Women (PTW) and Transgender Women (TW) in Lima, Peru using an explanatory sequential design where online surveys were administered followed by interviews. Based on the findings, practices and perspectives on HIV testing and treatment were compared quantitatively and qualitatively between PTW and TW participants.

--Firstly, I want to congratulate the authors for targeting an extremely hard to reach population (PTW) for some very timely research, since the global drive to end the HIV epidemic would not be successful without HIV prevention and care access for all hidden, vulnerable, hard to reach (and understand) populations like the TW and PTW.

--The article is well written, easy to follow, and the conclusions seem generally plausible given the results of the statistical analyses. However, I do have a few comments:

**Major Comments**

**1. The authors mentioned this as one of their limitations, but I wonder how much of the perceived differences in demographic characteristics between TW and PTW are driven by the difference in recruitment of the respective groups. TW were recruited from clinics and can potentially be more vulnerable than the average TW population in Lima, and PTW were recruited by the TW seeds, by virtue of the definition, were probably from a more general pool of PTWs in Lima.

Do the researchers have any data to support or refute that claim? For example, one metric might be HIV prevalence among recruited TW versus the general TW population, which seems to be similar in this case (30% in GP vs 26.1-37.7% in the study). Another important factor might be sex work status. Sex work is a known risk factor for HIV and other STIs as well and is known to be correlated with lower income and lower education. Can the researchers track the sex work status of all survey TW participants, and compare them with the TW population average in Lima? Any other metric to use perhaps?**

*Response:* Thank you for this comment.

Comparison to other TW: We would like to clarify that we did not recruit TW from clinics; TW were recruited at the “casa trans”, which are boarding houses for TW (see lines 87-89). In our discussion section, we note that the recruitment strategy could reduce the generalizability of these results to TW in settings outside of the casa trans. You are right that it is reasonable to believe that TW in *casa trans* may be more vulnerable than TW who do not live at such houses, as those in *casa trans* are often engaged in sex work, perhaps more than regular TW, but we do not have data to confirm this.

We do not believe this negatively impacts our study, as we are most interested in understanding the TW who live at the casa trans, as they are at high risk for HIV and easily accessible for prevention interventions.

Comparison between TW and PTW: You are right in that differences in the demographic characteristics between TW and PTW may be driven by differences in how they were recruited, but we have limited research to support this hypothesis.

- PTW were in the TW’s network, but they did come from a variety of places across Lima, whereas as TW were all based in the *casa trans*.
- We can assume many of the TW in this study were engaged in sex work, but we do not have data on if, or how regularly, the PTW in this study sold or purchased sex.
- We do know that HIV prevalence (by self-report) was substantially lower amongst PTW than TW in this study (Table 1); this could be true of the broader TW/PTW populations or it may only reflect the TW and PTW who are specifically connected to *casa trans*. Regardless, these populations connected to the *casa trans* are of most interest to us, as they are the most accessible for HIV prevention/treatment activities.

**2. Another limitation that the authors mention about the statistical analysis is the following:

‘Secondly, we did not adjust for clustering present from respondent driven sampling because this was an exploratory study and there is no gold standard for analysis for this sampling method.’

The authors are probably correct in pointing out that there is no gold standard method for analysis of RDS data in the literature as of yet, however, note that a typical RDS has multiple waves of recruitment with a few initial seeds, which makes statistical modeling quite challenging because of the hierarchy of the different waves. However, the study design pursued here only employed one wave of RDS, and thus the ensuing data can be conceived as clustered, where each cluster consists of a TW seed and the PTWs they recruited. The authors should at least look whether such clustering exists (with respect to outcomes in Tables S3 and S4), for example, one can estimate the intra-cluster correlation coefficient (ICC). If indeed the ICC is high, one can pursue adjusted t-tests/chi-square tests for clustered data or regression approaches like LME to see if the inference is any different from the unadjusted analyses.**

*Response:* Thank you for this comment. Before we address your comment, we feel it important to note two things:

- We envision this paper as a primarily descriptive analysis, thus the inferential statistical analyses run (t-test, chi-square) are secondary to the purely descriptive statistics we present.
- The qualitative results were much richer and more informative than the quantitative results in this study.

With these considerations in mind, we conducted a sensitivity analysis by running adjusted analyses for the t-tests. We have included the results of these in the supplemental file (S3). The results of the adjusted t-test analyses for HIV testing revealed very low ICC values, suggesting little similarities by cluster; there were no changes to our final results. The adjusted t-test analyses for HIV treatment (for which ‘n’ was incredibly small) showed that there were more differences due to clustering, but there were still no differences to the interpretation of the results.

We have updated the language in our manuscript to reflect these additional analyses.

- In the ‘Analysis’ section of the Methods, we have added a paragraph to describe this sensitivity analysis (see lines 155-161). It reads as follows:

*Respondent driven sampling lacks a gold standard method to account for clustering in the analysis (23), but as our study used only one round of invitations (i.e., TW reaching out to their partners),* we conducted a sensitivity analysis in R 4.1 (R Core Team, Vienna, Austria), using routine methods to adjust for clustering (packages “lme4”, “lmerTest”, “CR2”, and “performance”)*. We did this for two sets of outcomes: difference in mean responses for TW and PTW preferences for (1) HIV testing and (2) HIV treatment. This analysis for HIV testing outcomes revealed very low ICC values, and there was no difference in significance for HIV testing or treatment outcomes. As sensitivity analyses did not alter interpretation of results, we did not conduct adjusted analyses for other outcomes.*

-In the ‘Limitations’ section of the Discussion, we have updated our language regarding the limitation of using the unadjusted analysis (Lines 368-372). This now reads as follows:

*Secondly, we did not adjust for clustering for all comparisons between PTW and TW as our sensitivity analysis of mean preferences for HIV testing and treatment between PTW and TW suggests adjusted analyses would not change interpretation of the results. Furthermore for “testing” outcomes, ICC values were very small, suggesting limited impact of clustering; for “treatment” outcomes, the impact of clustering appeared to be greater, but there were too few observations for any results to be significant in the adjusted or unadjusted analysis.*

***Minor Comments
1. Page 11, line 180, Table 1 – Median and IQR are probably better metrics for ‘Age’ than mean and SD.***

*Response:* Thank you for this suggestion. We have updated ‘age’ in Table 1 with median and IQR instead of mean/SD and range. The interpretation of the data has not changed as a result.

**2. Page 13, line 197, Table 2 – Not fully visible for review. A third of the table seemed to be cut-off, probably that page needs to be in landscape format.**

*Response:* Thank you for this comment. It is our understanding that PLoS asks all authors to include their tables in portrait format, even if they do not fit appropriately on the page. The full table can be seen in the “Draft” view of the document. For your convenience, I have pasted the table below.

**Reviewer #3**: The authors evaluated practices and preferences for HIV services in partners of TW, a highly at-risk hidden population.

The paper is clearly written and only minor edits are needed.

For readers who are not familiar with the Peruvian health system and current standard of care, I consider the paper could benefit from the following changes/additions:

1. **The authors described in the discussion that PrEP was implemented in Peru in June 2023. Since this question arose at the time of the results, I suggest moving this explanation to the introduction.**

*Response:*  Thank you for this suggestion. We have added this information to the Introduction to include this sentence:

*The need to elucidate how to best reach PTW is especially urgent, as the inclusion of PrEP to Peru’s national HIV prevention guidelines in June 2023 ushers in opportunities to offer HIV prevention services to both TW and PTW (18), which are overdue*. (Lines 69-71).

2. On page 3, line 17, the authors mentioned “non-traditional HIV testing options**”. I suggest adding in the introduction a short explanation about the standard of care for HIV testing in Peru and clarifying what are the “non-traditional HIV testing options” available.**

*Response:* Thank you for this note. We have now specified what is meant by “traditional” and “non-traditional” testing options in the introduction (lines 63-65). This now reads as follows:

*Traditional HIV testing, prevention, and treatment services in Peru are offered to anyone through the national health system at CERITS (government-run STI clinics) and there has been substantial outreach to reach TW through non-traditional services (community-based or venue-based testing) (16,17).*

*
