# Supplementary material for: Practices and preferences for HIV testing and treatment services amongst partners of transgender women in Lima, Peru: An exploratory, mixed methods study
Source: PLoS One. 2024 Jul 9;19(7):e0306852. doi: 10.1371/journal.pone.0306852 (PMC11232998; doi:10.1371/journal.pone.0306852)
Supplement: Attachment — Submitted filename: PLOS_ResponseToEditor_April8.docx [file pone.0306852.s008.docx]

**Response To Academic Editor**

**1. Choose either an English or Spanish abstract.**

*Response*: Thank you for your comment. We have removed the Spanish abstract from the submission.

**2. Even though you've discussed the transgender situation globally, please clarify the correlation between your topic and the research question in the introduction.**

*Response:*  Thank you for your observation and this suggestion. We have modified the wording in the introduction section to better highlight the connection between the topic (HIV testing and prevention services amongst PTW) and the research question at hand. Most prominently, we have adjusted the first sentence of the paper to include mention of the partners of transgender women to emphasize to the readers that partners of transgender women (PTW), not transgender women, are the focus of this paper.

The research question for this paper is: What are the practices and preferences for HIV testing and treatment amongst the sexual partners of transgender women in Lima, Peru? This is included in statement form in the manuscript in lines 49-52, where we define the goals of this study: “*We therefore conducted this study to describe the practices and preferences that PTW in Peru have for HIV testing and treatment services and to determine how such practices and preferences (1) compare to those of TW and (2) compare amongst PTW based on the type of relationship they have with TW.”*

We have described the importance of understanding the preference of HIV testing amongst partners of transgender women in lines 25-48 in the Introduction section. In particular, we have described the need to examine this issue in Peru in lines 34-48.

**3. I suggest revising the aim of this study to articulate how this research will make a significant contribution.**

*Response:* Thank you for this suggestion. We have modified our statement about how this research will make a significant contribution in lines 52-53. This now reads: *“These results can be used to tailor HIV prevention and care programs to best reach PTW, including those that focus on PrEP initiation and continuation.”*

In addition, we have added a sentence to the conclusion to highlight the importance of this work (see line 365): “*These results should be considered when designing HIV testing and prevention programs for PTWs.”*

**4. It appears you are using a mixed-methods approach; please be more specific in the data analysis section.**

*Response.* Thank you for this comment.

- Per best practice, we have included our study design in the title of this study (“an exploratory, mixed methods study”).
- We have followed best practices to describe the mixed methods design in this study in the first paragraph of the methods section (under study design, see lines 57-61). Here we state that we used an explanatory sequential design (QUAN🡪QUAL). Per best practices for reporting qualitative data, we have now also clarified that data integration happened through merging of the data: “We analyzed surveys and interviews separately, then merged the findings, using the qualitative data to explain the quantitative survey results.” (Lines 60-61).
- We have also clarified this in the ‘data analysis’ section of the results, specifically calling out “merging” as the form of data integration. This section in our manuscript now reads: “Data integration occurred through merging qualitative and quantitative results, with the qualitative findings used to explain and expound on key findings from the quantitative survey.” (Lines 144-145)

As reference, below is a document that describes some of the practices for mixed methods research:

NIH Office of Behavioral and Social Sciences. (2018). Best practices for mixed methods research in the health sciences (2nd ed). Bethesda: National Institutes of Health. Accessed via <https://implementationscience-gacd.org/wp-content/uploads/2020/11/Best-Practices-for-Mixed-Methods-Research-in-the-Health-Sciences-2018-01-25-1.pdf>

**5. The table 1 presents a detailed breakdown of the characteristics of study participants, including age, education level, nationality, employment status, income, sexual attraction, number of sex partners, and self-reported HIV status, differentiated between TW (transgender women, n=69) and PTW (presumably non-transgender participants, n=165). regarding Education and Employment: Could the authors delve into the factors contributing to the educational and employment disparities observed, particularly the barriers TW face in these areas?**

*Response:* Thank you for this thoughtful comment. We agree that it is interesting to consider the differences between TW and PTW. Unfortunately, delving into the specifics of these differences goes beyond the scope of our paper and involves much speculation. While we do not think it is the place of this study to specifically explore this difference, we do provide some speculation as to why these differences exist: the power differential between cis male PTW and TW (see lines 300-307). We use our qualitative findings, and past studies, to support this hypothesis.

We have rephrased this discussion in lines 301-305 to read as follows: *“Power differences between TW and their partners have been well-noted in the literature, largely due to cis males being in the greatest position of power in many societies [1,3,7,10]. This power differential between largely cis-gender male PTW and TW was observed in our survey findings, as PTW appeared to be of higher socioeconomic status than TW, evidenced by PTW generally having higher levels of education, employment, and income than their TW partners.”*

**6. How does the cultural and national context influence the study's findings, especially regarding social support systems and healthcare access?**

*Response:*  Thank you for this question. We have provided discussion on how the local cultural context influences findings in the discussion section. For example:

- In the second paragraph of the discussion (Lines 292-307) ,we discuss (1) how local culture could have made the Likert scales used in the study ineffective, thus limiting what we could conclude from our survey, and (2) the power differential that likely exists between PTW and TW due to the local context, and how this could affect their access to health care.
- In the fourth paragraph of the discussion, we discuss specific recommendations for improving access to HIV treatment services: decreasing wait time and strengthening sensitization for healthcare workers on patient privacy and transgender care. These are both directly related to the local context of how HIV testing services are delivered in Peru. We have added new additional language (and additional citations) into the text to make note of the influence local context has on this issue (Lines 322-324).

*“These suggestions speak to the specific challenges of accessing and interacting with the local health system in Lima and align with key challenges noted in the literature, namely an under resourced primary health care system and the need to address stigma surrounding HIV, including in the healthcare setting (28–31).”*

**7. The authors are invited to explore how the sexual attraction and partner preferences among TW and PTW inform our understanding of HIV risk and prevention needs within these communities.**

*Response:*  Thank you for this suggestion. We agree that this is an important topic of research. Unfortunately, the discussion of partner preference and risk is outside the scope of our current study, which seeks to describe the practices and preferences that PTW in Peru have for HIV testing and treatment services.

However, we have addressed this in our past research, which we cite in this paper. For more information on this topic, please see these other papers published by our research group (which we have also referred to in this manuscript):

Long JE, Montaño M, Sanchez H, Huerta L, Calderón Garcia D, Lama JR, et al. Self-Identity, Beliefs, and Behavior Among Men Who Have Sex with Transgender Women: Implications for HIV Research and Interventions. Arch Sex Behav. 2021; 50(7):3287–95.

Long JE, Ulrich A, White E, Dasgupta S, Cabello R, Sanchez H, et al. Characterizing Men Who Have Sex with Transgender Women in Lima, Peru: Sexual Behavior and Partnership Profiles. AIDS Behav. 2020; 24(3):914–24.

Long JE, Sanchez H, Dasgupta S, Huerta L, Calderón Garcia D, Lama JR, et al. Exploring HIV risk behavior and sexual/gender identities among transgender women and their sexual partners in Peru using respondent-driven sampling. AIDS Care. 2022;34(9):1187–95.

Long JE, Sanchez H, Dasgupta S, Huerta L, Garcia DC, Lama JR, et al. Self-Reported Knowledge of HIV Status Among Cisgender Male Sex Partners of Transgender Women in Lima, Peru. J Acquir Immune Defic Syndr. 2022 May 1;90(1):1–5.

Long JE, Tordoff DM, Reisner SL, Dasgupta S, Mayer KH, Mullins JI, et al. HIV transmission patterns among transgender women, their cisgender male partners, and cisgender MSM in Lima, Peru: A molecular epidemiologic and phylodynamic analysis. Lancet Reg Health Am. 2022; 6.

**8. Given the marked HIV status disparity, what specific, actionable recommendations do the authors propose to mitigate this risk among TW?**

*Response:*  We appreciate this question. The goal of this paper was to describe the practices and preferences for HIV prevention amongst partners of transgender women. TW were not the focus of our paper. We did not conduct HIV testing in this study, or compare the prevalence of HIV amongst TW, PTW, or the broader population, so we believe proposing actionable research to mitigate the risk of HIV amongst TW is outside the scope of this paper.

**Response To Reviewer**

**Reviewer #2: The authors have done an excellent job addressing all the comments left by the Reviewers. I just have one minor comment that is mainly about consistency in presentation of results.**

- **In Supplementary Tables S3 and S5 (and possibly others), the authors have presented standard deviations (I presume for the data distribution), however, given they present results from hypothesis tests conducted on the means, the more relevant metric would be standard errors for these parameters. Can you present standard errors instead of SDs (or in addition to the SDs) in the Tables?**
- **Additionally, to be consistent, can you please add the cluster-adjusted standard errors in Supplementary Table S3-2?**

*Response:*

Thank you for this note. This was a descriptive study, so we are describing the sample population in all the data presented in the supplemental tables. We are not seeking to make inference on the overall population.

As standard deviations are used to describe the sample, while standard errors are used when making inference on the broader population, we believe that inclusion of standard deviation, not standard errors, is the most appropriate choice for these tables.

We have uniformly presented means and standard deviations in the relevant supplemental tables (S3 & S5).

*Relevant sources:*

[Nagele](https://pubmed.ncbi.nlm.nih.gov/?term=Nagele+P&cauthor_id=12644429) P. Misuse of standard error of the mean (SEM) when reporting variability of a sample. A critical evaluation of four anaesthesia journals. *Br J Anaesth.* 2003. 90(4): 514-6.  [doi: 10.1093/bja/aeg087](https://pubmed.ncbi.nlm.nih.gov/12644429/)

Kim H-Y. Statistical notes for clinical researchers: Understanding standard deviations and standard errors. *Restor Dent Endod*. 2013. 38(4): 263-265. doi: [10.5395/rde.2013.38.4.263](https://doi.org/10.5395%2Frde.2013.38.4.263)
